# Analysis of risk factors for surgical site infection after colorectal surgery: a cross-sectional study in the east of China pre-COVID-19

**DOI:** 10.3389/fpubh.2023.1204337

**Published:** 2023-08-10

**Authors:** Hui Sun, Hua Jiang, Zhi-Wei Jiang, Ge Fang, Zheng-Xiang Dai, Zhiguo Wang, Xiang Sun, Wen Wang

**Affiliations:** ^1^Medical Department, Affiliated Hospital of Nanjing University of Traditional Chinese Medicine, Nanjing, Jiangsu Province, China; ^2^Infection Management Department, Affiliated Hospital of Nanjing University of Traditional Chinese Medicine, Nanjing, Jiangsu Province, China; ^3^Department of Expanded Program on Immunization, Jiangsu Provincial Center for Disease Control and Prevention, Nanjing, Jiangsu Province, China; ^4^Department of Rheumatology and Immunology, The Affiliated Suqian First People’s Hospital of Nanjing Medical University, Suqian, Jiangsu Province, China

**Keywords:** surgical site infection, colorectal surgery, risk factor, prevalence, cross-sectional study

## Abstract

**Background:**

The occurrence of surgical site infection (SSI) can prolong the postoperative hospital stay, increase the economic burden of patients, and even endanger their lives. The purpose of this study was to investigate the incidence, risk factors, and microbiology of SSI after colorectal surgery (CRS) and to provide a basis for the prevention and control of SSI.

**Methods:**

A single-center, prospective, cross-sectional study of adult patients undergoing CRS was conducted from 2010–2019. Univariate and multivariate logistic regression models were used to collect and analyze demographic information, hospital characteristics, and potential perioperative risk factors of SSI.

**Results:**

A total of 3,302 eligible patients were included in this study, with 213 cases experiencing SSIs, resulting in an infection rate of 6.45%. Notably, the incidence of SSI decreased from 13.33% in 2010 to 3.56% in 2019 (P_trend_ < 0.001). *Escherichia coli* accounted for the majority of isolated microorganisms (37.09%), with 49 strains exhibiting resistance to one or more antibiotics (35.25%). Multivariate analysis showed that diabetes, anastomosis leakage, wound classification (contaminated/dirty), operation duration, blood loss greater than 200 ml, and NNIS risk index score for 2 or 3 were independent risk factors. Conversely, laparoscopic approach, preoperative bowel preparation and preoperative albumin levels emerged as protective factors against SSI after CRS. Furthermore, compared to non-SSI patients, SSI patients had a significantly higher 30-day mortality rate following surgery (0.23% vs. 2.35%, *p* < 0.05).

**Conclusion:**

SSI after CRS was susceptible to many factors, and the pathogen of SSI was mainly *Escherichia coli*. In clinical practice, measures such as correcting preoperative hypoproteinemia, choosing laparoscopic surgery, preoperative bowel preparation and shortening the duration of surgery should be taken to reduce the incidence of SSI.

## Introduction

Healthcare-associated infections (HCAIs) refer to infections that occur in patients during the process of care in hospitals or other healthcare facilities (1). These infections mostly occur three or more days after admission, and HCAIs are currently a major public health issue that requires monitoring and control by health departments in various countries. SSIs remain one of the most common types of HCAIs, and according to research by European scholars, SSIs are the main cause of HCAIs (19.6%) (2). The occurrence of SSIs is accompanied by prolonged hospitalization and a higher risk of death. Due to the large number of microorganisms in the rectum and colon, the incidence of postoperative incision SSI is higher in CRS compared with surgery on other parts of the body, but the difference is large (often 10–30%) (3–9). In addition, SSIs also result in a significant increase in economic burden. It is estimated that 157,000 patients in the United States experience SSI each year, resulting in an additional cost of approximately $3.3 billion (10).

SSIs are typically caused by an imbalance between bacteria and the body’s defense, and are related to many endogenous and exogenous factors (11, 12). The most important related factors may include surgical type, degree of contamination, age, length of surgery, comorbidities, and American Society of Anesthesiologists (ASA) classification (13, 14), etc. The presence of a large number of microorganisms in the CRS may cause contamination of the wound, resulting in a particularly high incidence of SSIs. Some studies have found that measures such as maintaining normal blood pressure and blood glucose levels, and appropriate use of antibiotics can reduce the incidence of SSIs (15), while other similar studies have not yielded the same results (16). Therefore, in the population undergoing colorectal surgery, the contribution of patient characteristics needs to be further considered (17–19).

There were international guidelines for the prevention of SSIs in many regions of the world (20–24). A well-performed SSI surveillance system can effectively reduce its incidence and was critical to reducing the burden of infection as well as understanding patient characteristics. Health departments in many countries, including China, are dedicated to developing infection control systems. The US Centers for Disease Control and Prevention’s National Nosocomial Infections Surveillance (NNIS) system can compare the incidence of SSIs among different hospitals, but we still need to understand patients and other risk factors. To this end, we conducted a prospective, single-center, cross-sectional study on the incidence of SSIs and related risk factors, as well as the distribution of pathogens after CRS, at a tertiary hospital in Jiangsu Province, China. our study contributes novel and innovative insights into the prevention, microbiology, risk factors, and outcomes associated with SSIs following colorectal surgery. The comprehensive analysis of a large patient cohort, coupled with the examination of temporal trends and identification of risk and protective factors, enhances our understanding of this complex surgical complication. These findings have important implications for clinical practice, infection control strategies, and patient care, highlighting the significance of our research for the scientific community and the broader healthcare system.

## Methods

### Study design

A single-center, prospective, cross-sectional study of adult patients undergoing CRS was conducted from January 1, 2010 to December 31, 2019. The follow-up period was defined as 30 days after surgery. The single-center was a tertiary general hospital, the patients were identified using an inpatient SSI Surveillance System database and hospital Information Systems (HIS), and the data on the basic characteristics of patients were collected prospectively. After obtaining approval from the Ethics Review Committee of the Nanjing University of Chinese Medicine Affiliated Hospital, we conducted a retrospective analysis of the data. All patients who underwent colorectal surgery were included in the study, while patients under the age of 18 and deceased patients were excluded.

### Data collection

Standardized questionnaires were used to collect information, and all enrolled patients were required to sign a written informed consent before participating in the study. The obtained data includes patient information before, during, and after surgery, with the following data list (1): Basic information (name, age, gender, body mass index (BMI), diabetes, hypertension, etc.) (2); Surgical situation (preoperative: hemoglobin (HB), albumin (ALB), antibiotic use, ASA grade (1–5, or), etc.; intraoperative: surgical method, wound classification (clean or I, clean/ contaminated or II, contaminated or III, and dirty or IV), ways of bowel preparation (oral antibiotics bowel preparation [OABP] with mechanical bowel preparation [MBP], MBP without OABP, none), operation time, etc.; postoperative: diagnosis and classification of SSI (superficial incisional, deep incisional, or organ/space), NNIS risk index(the NNIS risk index represented an internationally recognized stratification method for surgical risk. The NNIS risk index ranges from 0 to 3 and is determined by evaluating factors such as surgical duration, surgical wound classification, and ASA score. Each variable had critical thresholds: a contaminated or dirty surgical incision, a surgical duration of 225 min, and an ASA score of 3. If any of these variables exceed their critical threshold, a score of 1 is assigned.), information on isolated pathogens and antibiotic susceptibility, etc.).

### Primary outcome

The primary outcome was the incidence of SSI within 30 days after surgery. SSI was classified according to the Centers for Disease Control and Prevention (CDC) criteria as superficial incisional (Superficial incisional infection occurs within 30 days after surgery and only affects the skin or subcutaneous tissue of the incision), deep incisional (Deep incisional infection occurs within 30 days after surgery for patients without implants and within 1 year after surgery for patients with implants), and organ/space infections (Organ/space infection occurs within 30 days after surgery for patients without implants and within 1 year after surgery for patients with implants) (25, 26). The follow-up period was 30 days after surgery, and if the patient was discharged, follow-up was conducted by phone. However, SSI was diagnosed primarily by physical exam findings documented by the operating surgeon.

### Statistical analysis

All data were analyzed by SPSS software (version 22). Non-normally distributed continuous variables were expressed as M (interquartile spacing) and statistical analysis was performed using the Mann–Whitney U test. Fisher’s exact test or Chi-square test were used to compare the qualitative data and the logistic regression models were used to analyze the risk factors for SSI, and factors with *p* < 0.05 in the single-factor analysis were included in the multifactor analysis. *p* < 0.05 were considered significant.

## Result

### The incidence of SSI after CRS, 2010–2019

**Figure 1:**
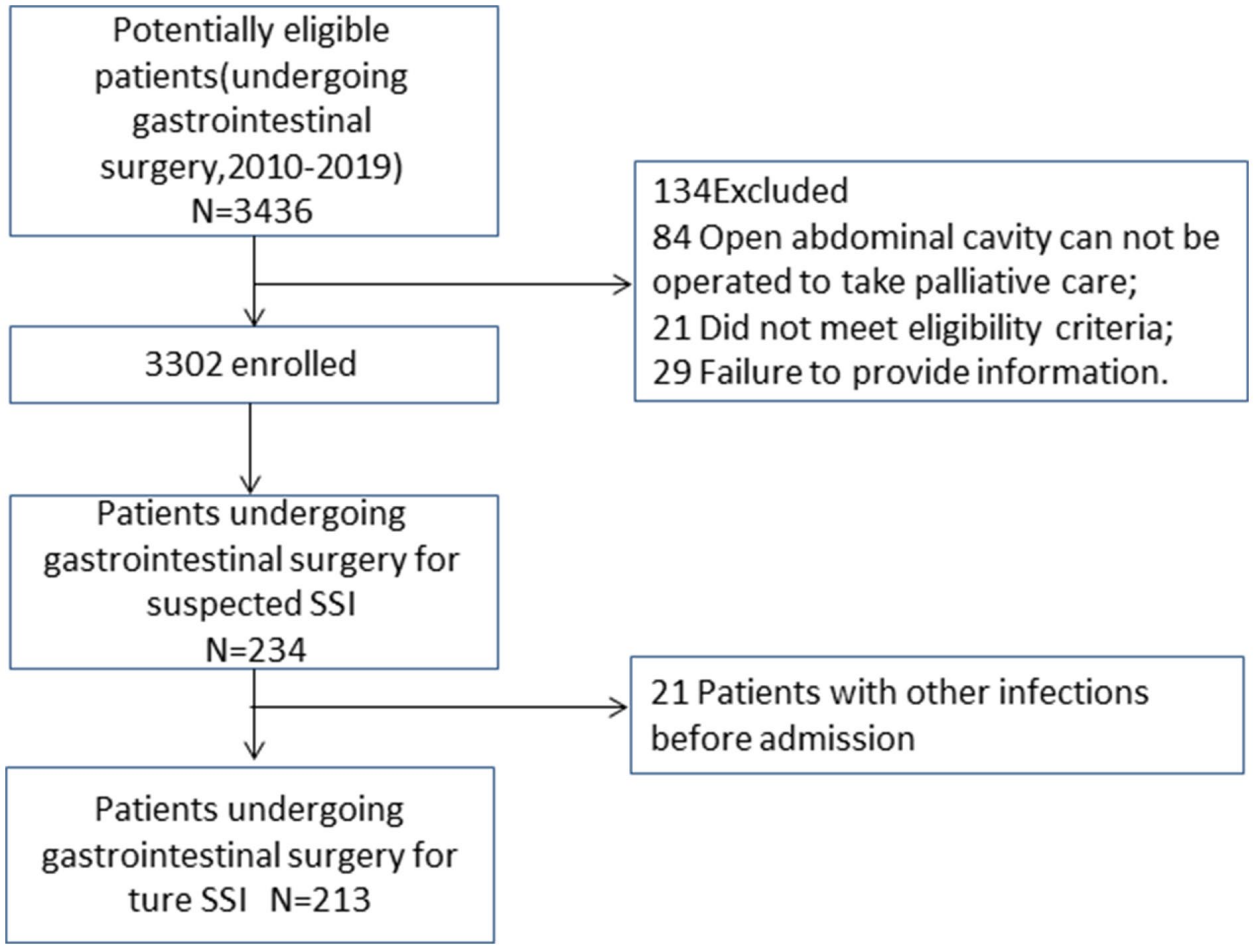
Flowchart of the study population.

**Figure 2:**
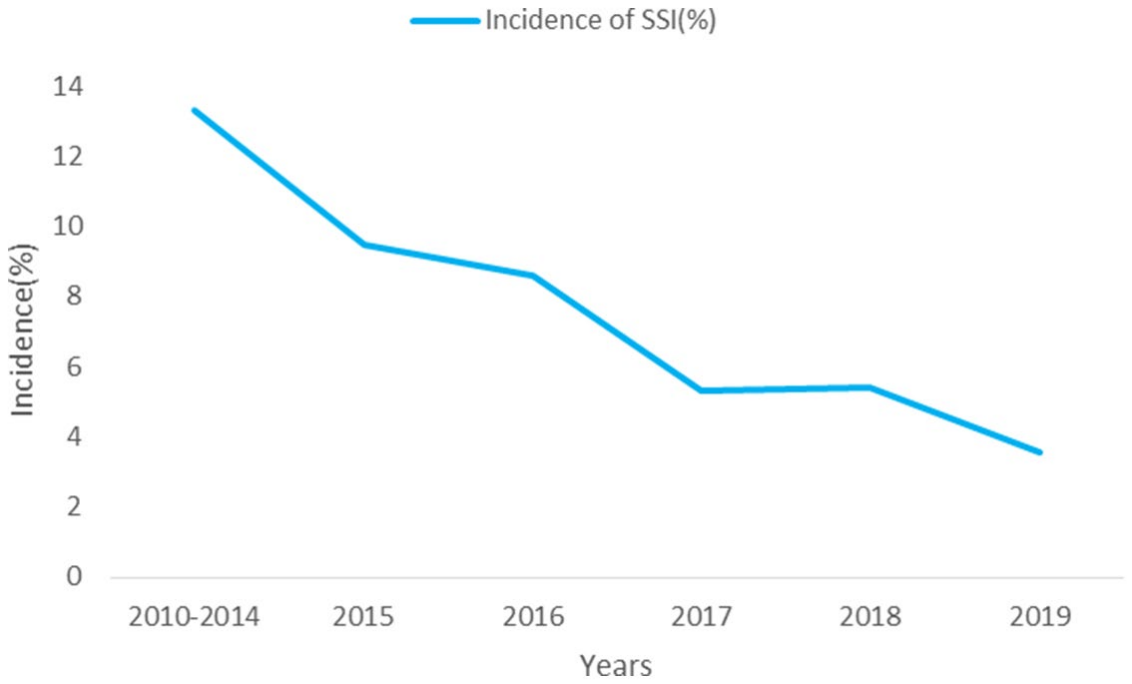
The incidence of SSI, 2010–2019.

This study included 3,302 eligible patients (Figure 1), of whom 65.2% were male, with a median age of 64 (15) years and a BMI of 23.2 (4.3) kg/m2. A total of 213 patients developed SSI (Figure 1), with an infection rate of 6.45%. The incidence of SSI decreased from 13.33% in 2010 to 3.56% in 2019 (P_trend_ < 0.001; Figure 2). Among them, 135 cases (4.09%) were superficial SSIs, 43 cases (1.41%) were deep SSIs, and 35 cases (1.06%) were organ/space SSIs (Table 1).

**Table 1:** The incidence of SSI after CRS.

	Cases, No. (%)	Incidence of SSI (%)			Total
		Superficial	Deep	Organ/lacunar	
Rectal	1,279 (38.72)	27 (2.11)	8 (0.63)	8 (0.63)	43 (3.36)
Colon	2023 (61.28)	108 (5.34)	35 (1.73)	27 (1.33)	170 (8.39)
Total	3,302 (100.00)	135 (4.09)	43 (1.41)	35 (1.06)	213 (6.45)

### Analysis of risk factors for SSI after CRS

Univariate analysis showed no significant differences between the non-SSI group and the SSI group in terms of gender, age, smoking, drinking, and other factors. However, there were statistically significant differences in 11 variables between the two groups. Furthermore, multivariate analysis showed that diabetes was the most common comorbidities associated with SSI [OR = 2.19 (1.42–3.37)]. Laparoscopic approach, preoperative albumin level and preoperative bowel preparation were protective factors for SSI after colorectal surgery (*p* < 0.001). Anastomosis leakage, wound classification(contaminated or dirty), operation lasted over 120 min, blood loss greater than 200 ml, and NNIS risk index score for 2 or 3 were independent risk factors for SSIs after CRS (Table 2).

**Table 2:** Univariate and multivariate analysis of risk factors in patients with SSI.

Variable	Category	Non-SSI group	SSI group	Univariateanalysis		Multivariate analysis	
				*U/x* ^2^	*p*	OR(95%CI)	*p*
Age [year, M (IS)]		64 (15)	66 (17)	309910.5	0.156		
Gender [N(%)]	Male	2006 (93.2)	147(6.8)	1.458	0.227		
	Female	1,083 (94.3)	66 (5.7)				
BMI [N(%)]	<24	1817 (94.4)	107 (5.6)	7.301	0.007	1	0.452
	≥24	1,229 (92.1)	106 (7.9)			1.14 (0.81–1.63)	
chronic disease [N(%)]	No	1886 (94.7)	105 (5.3)	11.470	0.001	1	0.106
	Yes	1,203 (91.8)	108 (8.2)			1.34 (0.94–1.91)	
diabetes [N(%)]	No	2,704 (94.2)	167(5.8)	14.661	0.000	1	0.000
	Yes	385 (89.3)	46 (10.7)			2.19 (1.42–3.37)	
Smoking [N(%)]	No	2078 (93.3)	149 (6.7)	0.648	0.421		
	Yes	1,011 (94.0)	64 (6.0)				
Drinking [N(%)]	No	2,493 (93.4)	176 (6.6)	0.473	0.492		
	Yes	596 (94.2)	37 (5.8)				
Operative Approach [N(%)]	Open	1,498 (90.2)	162 (9.8)	60.477	0.000	1	0.000
	Laparoscopic	1,591(96.9)	51 (3.1)			0.34(0.23–0.51)	
Wound class [N(%)]	clean	1,297 (96.2)	51 (3.8)	26.818	0.000	1	0.027
	contaminated/dirty	1792 (91.7)	162 (8.3)			1.65(1.06–2.57)	
Preoperative chemotherapy [N(%)]	No	2,745 (93.8)	181 (6.2)	3.296	0.069		
	Yes	338 (91.4)	32 (6.5)				
Bowel preparation [N(%)]	None	7 (36.8)	12 (63.2)	101.893	0.000	1	
	MBP+ OABP	18 (94,7)	1 (5.3)			0.01 (0.00–0.10)	0.000
	MBP	3,064 (93.9)	200 (6.1)			0.04 (0.01–0.12)	0.000
Anastomosis leakage [N(%)]	No	3,021 (96.6)	105 (3.4)	932.107	0.000	1	0.027
	Yes	68 (39.0)	108 (61.0)			39.01 (26.13–58.24)	
Operative time [N(%)]	<120 min	1,156 (95.8)	51 (4.2)	15.662	0.000	1	0.000
	≥120 min	1931 (92.3)	162 (7.7)			2.31 (1.49–3.59)	
ASA [N(%)]	1	95 (92.2)	8 (7.8)	0.306	0.580		
	2–3	2,994 (93.6)	205 (6.4)				
NNIS [N(%)]	0–1	2,520 (95.2)	128 (4.8)	57.720	0.000	1	0.015
	2–3	569 (87.0)	85 (13.0)			1.71 (1.11–2.64)	
Blood loss [N(%)]	<200 ml	2,454 (94.9)	131 (5.1)	37.766	0.000	1	
	≥200 ml	635 (88.6)	82 (11.4)			2.04 (1.40–2.98)	0.000
Hospital stay[day, M (IS)]		16 (9)	17 (9)	323640.5	0.686		
ALB [g/L, M (IS)]		37.8 (5.7)	36.8 (7.0)	378872.0	0.000		
HB [g/L, M (IS)]		124 (30)	121 (29)	328013.0	0.877		

OR, odds ratio; CI, confidence interval; IS, Interquartile space.

### Analysis of pathogen distribution and antibiotic resistance in SSI patients

139 pathogens were isolated from the body fluids of 213 patients with SSI, including pus, sputum, and feces. Among them, *Escherichia coli* was detected in 79 cases (37.09%), followed by *Acinetobacter baumannii* in 21 cases (9.86%), and the detection rates of Klebsiella pneumonia and *Pseudomonas aeruginosa* were 7.51 and 5.63%, respectively. The proportion of cultures negative or without any pathogens was 34.74% (Table 3). Although 49 strains of bacteria were resistant to one or more antibiotics (35.25%, 49/139), multidrug-resistant organisms (MRSA) were rare.

**Table 3:** Distribution characteristics of pathogenic bacteria in SSI patients.

Pathogen	No. (%) of SSIs
*Escherichia coli*	79 (37.09)
*Klebsiella pneumoniae*	16 (7.51)
*Pseudomonas aeruginosa*	12 (5.63)
*Acinetobacter baumanii*	21 (9.86)
*Staphylococcus aureus*	7 (3.29)
*Proteus mirabilis*	2 (0.94)
*Enterococcus faecium*	1 (0.47)
*Enterobacter cloacae*	1 (0.47)
Negative or no cultures taken	74 (34.74)

### Comparison of primary outcomes after CRS

The follow-up data from 2010 to 2019 showed a total of 11 deaths within 30 days after surgery, including 5 cases (2.35%) in the SSI group and 7 cases (0.23%) in the non-SSI group, with a significant statistical difference between the two groups (*p* < 0.05).

## Discussion

Overall, we found 213 cases (6.45%) of SSI among 3,302 colon surgery patients. Compared to other studies (6, 27, 28), there were significant differences in the incidence of SSI, which may be due to differences in the definition of SSI and postoperative follow-up time. In addition, our results showed that the incidence of SSI after colon surgery decreased from 13.33% in 2010 to 3.56% in 2019, and there was a significant decrease in SSI incidence after CRS. According to the “Guideline for the Prevention and Control of Surgical Site Infection (Trial)” issued by the National Health Commission of the People’s Republic of China (NHCPC) (26), prevention of SSI should be carried out preoperatively, intraoperatively, and postoperatively, such as the use of prophylactic antibiotics, preoperative bowel preparation, temperature and blood glucose control. The hospital determines possible risk factors based on the analysis of actively monitored, and regularly provides feedback and summaries of SSI. The active monitoring and preventive control measures of SSI have played a significant role in reducing the incidence.

Previous studies have found that the occurrence of healthcare-associated infections mainly depends on the number of colonizing pathogens and the host’s immune response. Since anaerobic and gram-negative bacteria mainly colonize the rectum and distal ileum, surgery in the colon inevitably causes intestinal bacterial translocation, resulting in a higher incidence of SSI after colorectal surgery (29). In this study, a total of 139 pathogenic bacteria were isolated, with gram-negative bacteria accounting for more than 60% of the isolates, and *Escherichia coli*, *Acinetobacter baumannii*, *Klebsiella pneumoniae*, and *Pseudomonas aeruginosa* being the most common pathogens. It is noteworthy that 49 strains were found to be resistant to one or more antibiotics, and Emine et al. (30) had reported that the use of antimicrobial agents can reduce the incidence of surgical site infections, but the irrational use of antibiotics can lead to antibiotic resistance, and at the same time increase the risk of SSI.

Multifactorial analysis reveals that diabetes is the most prevalent comorbidity [OR = 2.19(1.42–3.37)]. Furthermore, univariate analysis indicates that chronic conditions (with a primary focus on hypertension in this study) are risk factors for the occurrence of surgical site infections (SSI) after breast cancer and cesarean section procedures (31, 32). Due to increased vascular resistance, hypertension patients are more likely to experience peripheral blood supply changes, thereby increasing the risk of tissue infections. This conclusion was consistent with domestic research, but further studies are needed to confirm our hypothesis as we did not record specific changes in blood pressure values. Diabetes is a metabolic disorder disease, and high blood sugar is conducive to the growth and proliferation of bacteria while also hindering wound healing (33, 34). In the multifactorial analysis, four variables were ultimately confirmed. Our results demonstrated that contaminated or dirty wound classification significantly increases the risk of CRS-associated SSI by 1.65 times when compared to clean wound classification, a finding consistently supported by numerous studies. Anastomotic leakage, a severe complication after rectal cancer surgery, can lead to serious intra-abdominal infections, causing patient distress, prolonged hospitalization, increased treatment costs, and in severe cases, even mortality. Our research also showed that compared to patients without anastomotic leakage, the risk increased by 39.01 times. Prolonged surgical time and increased intraoperative blood loss were independent risk factors for SSI development in CRS, likely due to prolonged exposure to pathogenic microorganisms, potentially leading to an increased risk of infection (29, 35, 36). Therefore, greater attention should be given to the perioperative prevention of SSI in patients with a history of incision contamination in clinical practice. Our results further indicated that NNIS risk index scores of 2 or 3 are independent predictors of SSI, consistent with previous research findings (37).

In recent years, laparoscopic surgery has rapidly gained popularity worldwide due to its small incision and fast recovery features. Laparoscopic surgery can significantly shorten hospitalization and normal intestinal function recovery time in CRS, reduce intraoperative bleeding, and to a certain extent, reduce the incidence of SSI (38–41). This study shows that the SSI incidence rate is only 3.1% in laparoscopic surgery, while it is as high as 9.8% in open surgery, and the difference between the two has statistical significance, which was a protective factor for SSI after CRS. In addition, albumin is a commonly used indicator to evaluate patients’ nutritional status. If a patient’s albumin level is low, it indicates malnutrition, which can be accompanied by tissue edema and decreased resistance, increasing the risk of postoperative complications (42–44). In our study, the preoperative albumin level in the NON-SSI group was higher than that in the SSI group [37.8(5.7) g/L vs. 36.8(7.0) g/L), and it had statistical significance (*p* < 0.05).

Preoperative bowel preparation remains a topic of great controversy and interest worldwide. The use of MBP and/or OABP has sparked numerous debates. Data collected from this study indicates that since 2014, our hospital has rarely used MBP and OABP. Nevertheless, multifactorial regression analysis still reveals that preoperative intestinal preparation serves as a protective factor against SSI in this research. The World Health Organization (WHO) recommends the adoption of preoperative MBP combined with OABP to reduce the risk of SSI in patients undergoing elective colorectal surgery (24). Due to the limited number of cases with combined preoperative MBP and OABP in this study, a further collection of more cases is needed for analysis.

Our study also had some limitations. Firstly, our study was based on data collected from a single center, which may introduce selection bias. However, it is worth noting that our research center was a prestigious tertiary medical institution located in the eastern region of China known as the Yangtze River Delta, where a considerable number of patients prefer to undergo colorectal surgeries. Secondly, the retrospective analysis of prospectively collected data could introduce bias and does not establish a definitive causal relationship between risk factors and SSI. Thirdly, similar to other comparable studies, our surveillance system database did not collect a sufficient number of risk factors for analysis. It is important to acknowledge that there may be other complications associated with deep or organ/space SSIs that were not accounted for in our study, which could have confounded the primary outcomes. Fourthly, the proportion of cultured samples from SSI patients in our study was relatively low, limiting the availability of comprehensive information on the causative pathogens involved. Lastly, this study lacks a longitudinal analysis, and it is impossible to understand the impact of relevant interventions on individuals by observing their changes at different time points.

## Conclusion

In summary, there were many factors that can affect the occurrence of SSIs after CRS. It was hoped that this will help healthcare professionals identify patients at higher risk of developing SSI after surgery, so that further strategies can be implemented to reduce the incidence of SSI. In clinical practice, measures such as correcting preoperative hypoproteinemia, choosing laparoscopic surgery, preoperative bowel preparation and shortening the duration of surgery should be taken to reduce the incidence of SSI.

## Data availability statement

The original contributions presented in the study are included in the article/supplementary material, further inquiries can be directed to the corresponding authors.

## Ethics statement

Written informed consent was obtained from the individual(s) for the publication of any potentially identifiable images or data included in this article.

## Author contributions

WW and XS conceived of this study. HS, HJ, Z-WJ, Z-XD and GF collected the data. HS, XS, and ZW analyzed the data and drafted initial manuscript. WW, XS, and ZW critically revised the manuscript and helped interpret data. All authors reviewed the final manuscript.

## Funding

This work was supported by the Jiangsu Health Development Research Center Open Project (JSHD2022043) and Jiangsu Provincial Geriatric Health Research Project (LKM2023005).

## Conflict of interest

The authors declare that the research was conducted in the absence of any commercial or financial relationships that could be construed as a potential conflict of interest.

## Publisher’s note

All claims expressed in this article are solely those of the authors and do not necessarily represent those of their affiliated organizations, or those of the publisher, the editors and the reviewers. Any product that may be evaluated in this article, or claim that may be made by its manufacturer, is not guaranteed or endorsed by the publisher.
